# Supplementary material for: Ultra-broadband local active noise control with remote acoustic sensing
Source: Sci Rep. 2020 Nov 27;10:20784. doi: 10.1038/s41598-020-77614-w (PMC7695846; doi:10.1038/s41598-020-77614-w)
Supplement: Supplementary file 1 — Supplementary Information. [file 41598_2020_77614_MOESM1_ESM.pdf]

# Ultra-broadband local active noise control with remote acoustic sensing – *Supplementary Information*

Tong Xiao[1], Xiaojun Qiu[1] & Benjamin Halkon[1]

[1]Centre for Audio, Acoustics and Vibration, University of Technology Sydney, Sydney, Australia. Correspondence and requests for materials should be addressed to T.X. (Tong.Xiao@student.uts.edu.au)

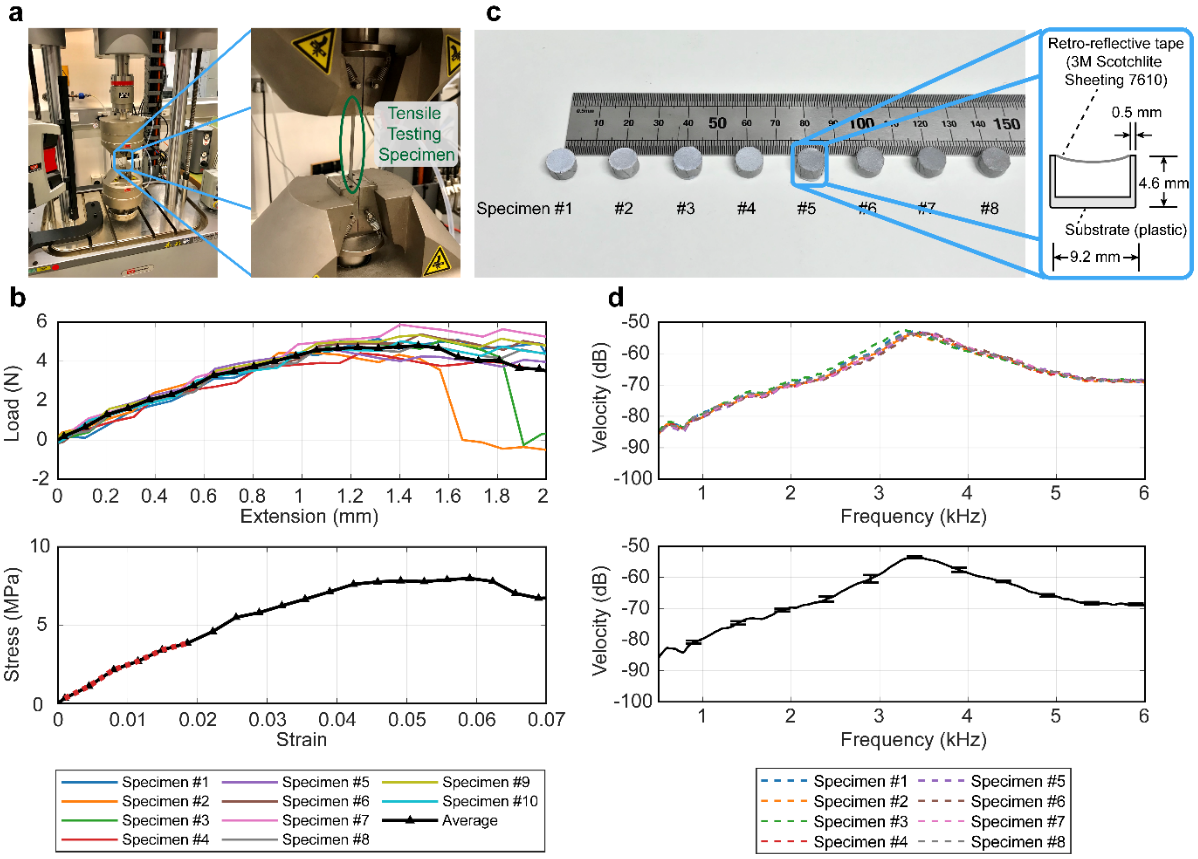


**Supplementary Figure S1**. (**a**) Photos of the tensile testing using an Instron ElectroPuls E10000 Test System to determine the properties of the retro-reflective material used in the system. Ten ASTM dog-bone specimens were cut out for the tensile testing. (**b**) Top – The load-extension curve of ten specimens from the tensile testing and the average value. Bottom – The stress-strain curve of the average value converted from the load-extension curve. The dotted part shows the elastic region of the testing material. (**c**) Eight nominally identical membrane specimens were used to determine the surface vibration frequency response performance. Membranes were placed in an environment within a semi-anechoic chamber and subjected to white noise excitation from a nearby loudspeaker. An LDV (Polytec PDV-100) was used to measure the resulting surface vibration from the centre of each membrane. (**d**) Top – The frequency response of the eight membrane specimens. Bottom – The mean value of the eight membrane specimens.

**Supplementary Table S1**. The measured parameter values of the retro-reflective material used in the system.

| Parameters | Symbol | Value | Unit |
|---|---|---|---|
| Thickness | $h$ | 0.1 | mm |
| Density | $\rho_M$ | 204 | $kg/m^3$ |
| Tension | $T$ | 33 | N/m |
| Young's modulus | $E$ | 205 | MPa |

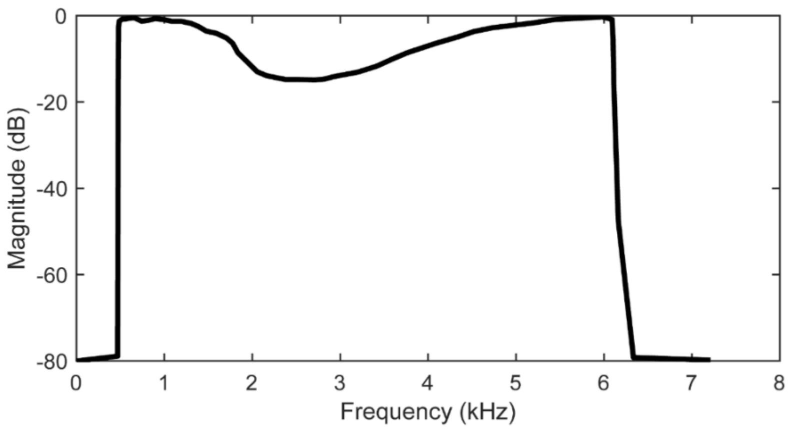


**Supplementary Figure S2**. The spectrum of a customised Fletcher-Munson curve filter

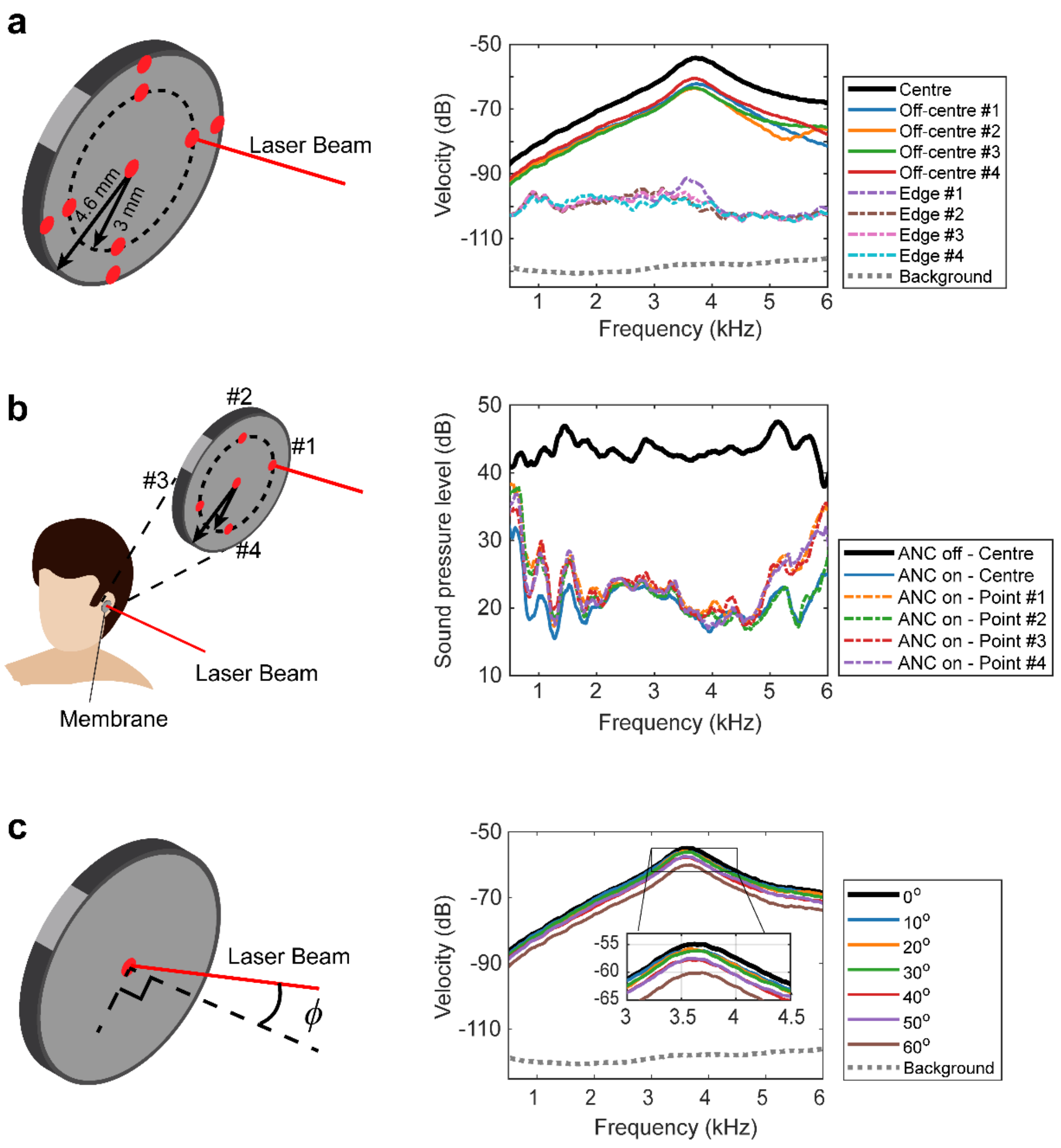


**Supplementary Figure S3**. (**a**) Locations of the test points on the membrane (centre, off-centre points #1 − #4, edge points #1 − #4) and the frequency responses at these points. (**b**) Locations of the test points on the membrane (centre, off-centre points #1 − #4) in the right ear of the HATS and the SPLs observed by the right ear of the HATS without ANC and with ANC using the LDV determined error signals at these points. (**c**) The incident laser beam from the LDV at an angle of $\phi$ with the normal direction and the corresponding frequency responses of the LDV signal with the incident angle from zero degrees to 60 degrees.

**Remarks on Supplementary Fig. S3**:

To understand whether the location of the probe LDV laser beam on the membrane pick-up significantly affects ANC performance, a series of tests were carried out by projecting the LDV (Polytec PDV-100) laser beam to different locations of the membrane as shown in Supplementary Fig. S3(a). A loudspeaker (Genelec 8010A) producing white noise was placed approximately 1.5 m away from the membrane in a hemi-anechoic chamber, with the membrane geometrical centre aligned with the loudspeaker axis. Supplementary Figure S3(a) also shows the frequency spectra of the LDV signal at the centre, four off-centre points and four edge points of the membrane. It is clear that the vibration velocity at the geometrical centre was the largest as expected. Although the magnitude decreased slightly, the velocity measured at the off-centre points can be used for ANC. At the edge of the membrane, there was a low vibration velocity level as expected due to the boundary constraints with such a measurement clearly not being of use as an ANC error signal.

It should be noted that the measurements at the edge were still slightly higher than the noise floor by about 15 dB. This was due to the vibration of the lightweight membrane as a whole body in the presence of the sound. In a related recent study[S1], the vibration velocity of the microphone diaphragm at multiple points along a line has been examined, and similar trends were observed.

With the exception of the edge, the LDV signals at the off-centre points on the membrane surface are still sufficient for ANC. To examine the ANC performance at these off-centre points, the membrane was placed in the head and torso simulator (HATS) to observe the change of the sound pressure level (SPL). The system was configured again similar to the reference[S2], where the primary source was placed about 1.2 m directly behind the HATS, and the right ear was controlled using the proposed method. Supplementary Figure S3(b) shows the SPL observed by the HATS without and with ANC. The control performances at the off-centre points were similar to that at the centre, albeit it with arguably reduced upper and lower frequency performance. This demonstrates that the ANC performance is not so sensitive to the precise location of the laser beam on the membrane, and similar ANC performance can be obtained when the laser beam is slightly off-centre. This is helpful since, during head-tracking, it

allows for some small lag between the membrane during motion and the probe laser beam which tracks it following the image-based algorithm which updates the galvanometer drive signals.

It is possible for membrane tilting to also be present and, to investigate this, different laser beam incident angles on the membrane surface were examined. The configuration of the testing system was the same as described previously. The incident angle of the laser beam varied at the centre of the membrane as shown in Supplementary Fig. S3(c). The frequency spectra illustrate that the LDV had the most significant response in the normal direction, as expected. As the incident angle increased, the magnitude of the response decreased, also as expected (since the LDV measures target surface velocity in the direction of the incident laser beam[S3]). The differences, however, were not dramatic. For example, when the incident angle was approximately 30 degrees, the magnitude dropped by approximately 1 dB, whereas when the incident angle was at approximately 60 degrees, the magnitude dropped around 5 dB. The ANC performance did not change significantly as a result of these differences.

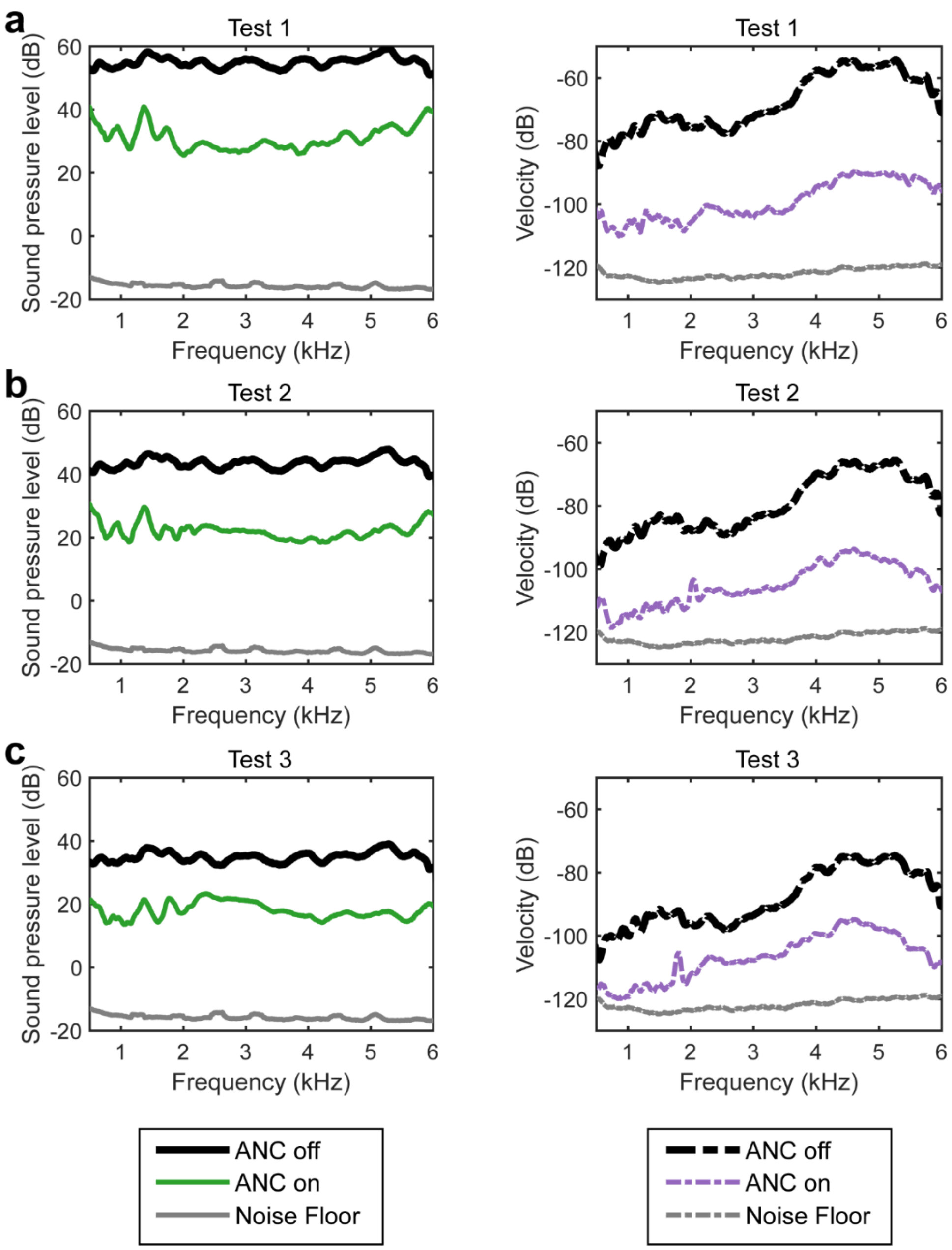


**Supplementary Figure S4**. The SPLs (left) and the magnitude of the LDV signal (right) without and with ANC for (**a**) test 1 where the SPL is 93.1 dB without ANC, (**b**) test 2 where the SPL is 81.8 dB without ANC and (**c**) test 3 where the SPL is 73.1 dB without ANC.

**Remarks on Supplementary Fig. S4**:

To examine the effects of the noise floor of the LDVs, different levels of the primary source signal have been studied. Similar to the reference[S2], where the primary source was placed about 1.2 m directly behind the HATS, the right ear was controlled using the proposed method. Three tests were performed with the grey noise described in the manuscript as the primary sound. These tests were carried out in a hemi-anechoic chamber to eliminate the interference from other sound sources. Note that the background SPL in the chamber is about 23 dBA. This background noise observed by the HATS is slightly higher than this value due to the ear simulators.

The ANC results of the error signals from the LDV and the SPLs observed by the HATS are shown in the Supplementary Fig. S4, and the corresponding overall values are shown in the Supplementary Table S2. It is clear that noise floor of the LDV signal is sufficiently low in these cases and doesn't cause any problem for the active noise control in this research. It is also interesting to note that ANC performance observed by the HATS in Test 2 is slightly better than that in the Fig. 4a in the manuscript. A possible reason is that the background noise in this case is lower than the one presented in the manuscript with a background noise of about 38.5 dBA.

**Supplementary Table S2**. The overall SPL and the LDV signal magnitude without and with ANC under three levels of sound.

| | **Overall SPL (dB)** | | | **Overall LDV Signal Magnitude (dB)** | | |
|---|---|---|---|---|---|---|
| | **Without ANC** | **With ANC** | **Reduction** | **Without ANC** | **With ANC** | **Reduction** |
| **Test 1** | 93.1 | 71.4 | 21.7 | −23.3 | −57.5 | 34.2 |
| **Test 2** | 81.8 | 61.7 | 20.1 | −34.7 | −63.0 | 28.3 |
| **Test 3** | 73.1 | 57.0 | 16.1 | −43.4 | −64.2 | 20.8 |
| **Noise Floor** | 34.3 | | | −83.8 | | |

**References**

S1. Suh, J. G., Cho, W. H., Kim, H. Y., Cui, Z. & Suzuki, Y. Sensitivity measurement of a laboratory standard microphone by measuring the diaphragm vibration. *Appl. Acoust.* **143**, 38–47 (2019).

S2. Elliott, S. J., Jung, W. & Cheer, J. Head tracking extends local active control of broadband sound to higher frequencies. *Sci. Rep*. **8**, 5403 (2018).

S3. Rothberg, S. J. et al. An international review of laser Doppler vibrometry: Making light work of vibration measurement. *Opt. Lasers Eng*. **99**, 11–22 (2017).

# Supplementary Movie 1 - Performance for a stationary head and torso simulator (HATS) with environmental noises

# Supplementary Movie 2 – The virtual ANC headphone with head tracking
